# The *bla_KPC-249_*-mediated mechanism drives the transition of ST463 *Pseudomonas aeruginosa* from Ceftazidime-Avibactam sensitivity to resistance during clinical treatment

**DOI:** 10.3389/fcimb.2026.1744386

**Published:** 2026-02-27

**Authors:** Xiaosi Li, Yan Feng, Xiaoyan Wu, Heping Shen, Shumi Shang, Wenting Tang, Fupin Hu, Huijun Liang

**Affiliations:** 1 Department of Laboratory Medicine, the Second Affiliated Hospital of Jiaxing University, Jiaxing, China; 2 Department of Laboratory Medicine, Heishui County People's Hospital, Aba Tibetan and Qiang Autonomous, Sichuan, China; 3 Department of Laboratory Medicine, Jiaxing Maternity and Child Health Care Hospital, College of Medicine, Jiaxing University, Jiaxing, China; 4 Department of Infectious Disease, the Second Affiliated Hospital of Jiaxing University, Jiaxing, China; 5 Institute of Antibiotics, Huashan Hospital, Fudan University, Shanghai, China

**Keywords:** *bla*_KPC-249_, Ceftazidime-avibactam, *P. aeruginosa*, resistance, ST463

## Abstract

**Introduction:**

Ceftazidime-avibactam represents a crucial therapeutic option for managing infections attributable to carbapenem-resistant *P. aeruginosa*. Nonetheless, the emergence of resistance to ceftazidime-avibactam in *P. aeruginosa* presents a significant challenge for clinical anti-infective therapy. This study primarily elucidates the mechanisms by which *P. aeruginosa* transitions from drug sensitivity to resistance during ceftazidime-avibactam treatment, ultimately resulting in therapeutic failure.

**Methods:**

The susceptibility testing was performed using the broth microdilution method, conjugation experiment was performed via the filter mating method, the genetic surroundings of *bla*_KPC-249_ and comparison of plasmids structures was performed using short/long-read genome sequencing and analysis method, the resistance of transconjugant carried the *bla*_KPC-249_ to ceftazidime-avibactam was performed via molecular cloning method.

**Results:**

For *P. aeruginosa* isolates from a patient, the minimum inhibitory concentrations (MICs) of ceftazidime-avibactam (CAZ-AVI) were determined as follows: isolates from sputum and bronchoalveolar lavage fluid both exhibited an MIC of 2 mg/L without blaKPC. In comparison, the blood-isolated strain P. aeruginosa PAE045 showed a significantly elevated MIC of >128 mg/L against CAZ-AVI. Plasmid conjugation experiments results demonstrated that the plasmid harboring the *bla*_KPC-249_ gene could be successfully transferred to the recipient strain PAO1*^rifR^* (rifampicin-resistant *P. aeruginosa* PAO1). Third-generation sequencing results revealed that the *bla*_KPC-249_ gene was located on a plasmid with an approximate size of 37 kb. Compared with the wild-type e *bla*_KPC-2_ gene, the *bla*_KPC-249_ gene had two additional amino acid residues in its encoded protein: threonine (Thr, T) at position 182 and serine (Ser, S) at position 183. Furthermore, the upstream and downstream regions of the *bla*_KPC-249_ gene were flanked by the insertion sequences ISK*pn6* and ISK*pn27*, respectively.

**Discussion:**

These mobile genetic elements may play a role in the capture and dissemination of the *bla*_KPC-249_ gene. The *bla*_KPC-249_ gene is identified as a novel mutant variant of the *bla*_KPC_ gene family, which mediates the resistance of *P. aeruginosa* to the antimicrobial agent ceftazidime-avibactam.

## Introduction

*P. aeruginosa* is an important pathogen responsible for both nosocomial and community-acquired infections, with a particular predilection for elderly patients, immunocompromised individuals, and those with severe infections (Hu et al., 2024). It is capable of inducing a broad spectrum of infectious diseases, including cystic fibrosis-related pulmonary infections, pneumonia, and bloodstream infections (Hawkey et al., 2018; Zhang et al., 2024). The drug resistance mechanism of *P. aeruginosa* is highly complex, exhibiting multidrug resistance (MDR) to multiple classes of antibiotics, such as carbapenems and cephalosporins, thereby posing significant challenges to clinical anti-infective treatment (Hu Y. et al., 2021). Notably, carbapenem-resistant *P. aeruginosa* (CRPA) has been designated by the World Health Organization (WHO) as a high-priority pathogen for which the urgent development of new antimicrobial agents is required (Tacconelli et al., 2018). According to the bacterial resistance surveillance data from CHINET (https://www.chinets.com/Data/AntibioticDrugFast) (China Antimicrobial Resistance Surveillance Network, available at www.chinets.com), the resistance rates of carbapenem-resistant *P. aeruginosa* (CR-PAE) to imipenem, meropenem, levofloxacin, aztreonam, and piperacillin-tazobactam were 94.9%, 77.2%, 48.8%, 46.2%, and 42.1%, respectively. In contrast, the resistance rates of CR-PAE to ceftazidime-avibactam and colistin were 19.6% and 6.0%, respectively.

Ceftazidime-avibactam (CZA) is a novel β-lactam/β-lactamase inhibitor combination preparation, which exhibits inhibitory activity against the production of extended-spectrum β-lactamases (ESBLs), AmpC β-lactamase, class A serine carbapenemases (e.g., *KPC*), and class D OXA-48-type carbapenemases (van Duin and Bonomo, 2016). Ceftazidime-avibactam was approved for clinical use in the United States and Europe in 2016 and obtained marketing approval in China in 2019. *In vitro* antimicrobial susceptibility testing results have demonstrated that ceftazidime-avibactam exhibits potent antibacterial activity against carbapenem-resistant *P. aeruginosa* (CR-PAE), including strains producing KPC-type carbapenemases. However, with the widespread use of ceftazidime-avibactam in clinical practice, *P. aeruginosa* strains resistant to this agent have emerged and spread globally. Existing studies have indicated that *P. aeruginosa* harbors multiple resistance mechanisms to ceftazidime-avibactam, including mutation-associated overexpression of Pseudomonas-derived cephalosporinase (PDC), alterations in bacterial efflux pump systems and outer membrane protein permeability, the production of Class B β-lactamases, as well as carbapenemase hydrolysis mediated by specific *KPC* subtypes (Papp-Wallace et al., 2020). In 2017, it was first reported that the *bla*_KPC-3_ gene mutation mediates the resistance of *Klebsiella pneumoniae* to ceftazidime-avibactam (Shields et al., 2017). Subsequently, *KPC* gene mutations that occur during ceftazidime-avibactam treatment have been associated with therapeutic failure, and such cases are primarily documented in Enterobacteriaceae species, including *Klebsiella pneumoniae* and *Escherichia coli (*Hernández-García et al., 2021). However, reports on ceftazidime-avibactam resistance in *P. aeruginosa* caused by *bla*_KPC_ mutations remain scarce. To date, a total of 271 *KPC* variants have been identified (https://www.ncbi.nlm.nih.gov/pathogens/refgene/); among these, only six variants (*bla*_KPC-31_, *bla*_KPC-33_, *bla*_KPC-87_, *bla*_KPC-90_, *bla*_KPC-113,_ and *bla*_KPC-201_) have been confirmed to be associated with CZA resistance in *P. aeruginosa (*Faccone et al., 2022; Lee et al., 2023; Yang et al., 2023; Hu et al., 2024; Zhou J. et al., 2025; Zhou L. et al., 2025*).*

This study reported a case of *P. aeruginosa* infection. During the treatment with ceftazidime-avibactam, the *KPC* gene produced by *P. aeruginosa* led to a drug-resistant mutation that mediated the bacteria’s resistance to ceftazidime-avibactam, resulting in treatment failure.

## Materials and methods

### Case presentation

**Figure 1:**
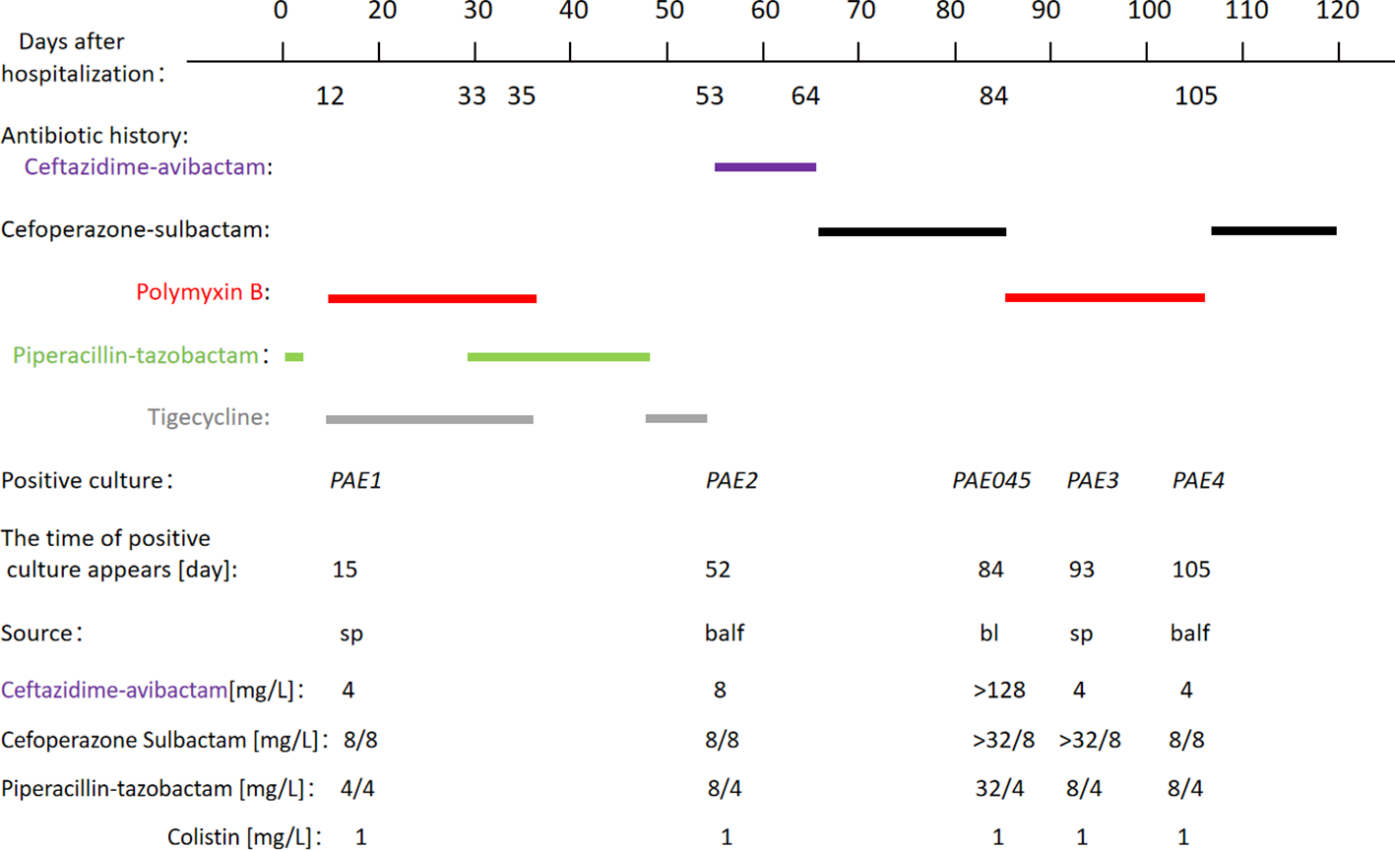
Antibiotic administration history and microbiology culture results. (The dosage regimens of the antimicrobial agents are detailed as follows: 4.5 g piperacillin–tazobactam, every 8 h, day 1 to day 12 days after admission; 0.5 million units polymyxin B, every 12 h, day 13 to day 35; 50 mg tigecycline, every 12 h, day 13 to day 35; 4.5 g piperacillin–tazobactam, every 8 h, day 33 to day 50; 50 mg tigecycline, every 12h, day 50 to day 53; 2.5g ceftazidime-avibactam, every 8 h, day 53 to day 64; 2 g cefoperazone-sulbactam, every 8 h, day 64 to day 84; 0.5 million units polymyxin B, every 12h, day 84 to day 105; 2 g cefoperazone-sulbactam, every 8 h, day 105 to day 120); PAE1 (2301150071), isolated on day 15; PAE2 (2302180062), isolated on day 52; PAE045 (2303241063), isolated on day 84; PAE3 (2303310085), isolated on day 93; PAE4 (2304140080), isolated on day 105; sp: sputum; balf: bronchoalveolar lavage fluid; bl: blood.

A 64-year-old woman was admitted to the Department of Respiration of a local hospital for pulmonary infection. The patient had been given anti-infective treatment with piperacillin-tazobactam at a dose of 4.5 g every 8 hours (q8h) for 12 days. On day 13, ABA(CRABA) was isolated from sputum culture, and the anti-infective regimen was adjusted to polymyxin B sulfate (500,000 IU, q12h, ivgtt) in conjunction with tigecycline (50 mg, q12h, ivgtt) for 11 days, until both lung exudates improved compared to before. Subsequently, the anti-infective regimen was adjusted to piperacillin-tazobactam (4.5 g, q8h) for 17 days. On day 50, the anti-infective regimen was switched to tigecycline (50 mg, q12h, ivgtt) for 3 days. On day 53, the inflammatory markers remained high, and correspondingly, the body temperature was poorly controlled; the anti-infective regimen was switched to ceftazidime-avibactam (2.5 g, q8h) for 11 days. On day 64, the patient was admitted to the ICU, whereupon the anti-infective regimen was switched to cefoperazone-sulbactam (2g, q8h) for 20 days. On day 84, PAE045 was isolated from blood culture obtained from the patient. In accordance with antimicrobial susceptibility testing findings, the anti-infective regimen was adjusted to polymyxin B sulfate, administered as 500,000 IU intravenously every 12 hours for 21 days. On day 105, the anti-infective regimen was switched to cefoperazone-sulbactam (2g, q8h) for 15 days, until both lung exudates had improved. (**Figure 1**). On day 178, the patient recovered uneventfully and was discharged from hospital.

### Clinical strains

*P. aeruginosa* strains PAE1, PAE2, PAE3, PAE4, and PAE045 were isolated from a 64-year-old female patient admitted to the Second Affiliated Hospital of Jiaxing University in China, between December 30, 2022, and June 26, 2023. Samples were collected from the patient’ lower respiratory tract and blood at various time intervals during treatment. All bacterial isolates were cultured on 5% sheep blood-supplemented Columbia agar for a duration of 18-24 hours at 35°C. *P. aeruginosa* isolates were validated by matrix-assisted laser desorption/ionization-time-of-flight mass spectrometry (MALDI-TOF MS system) (bioMérieux, France). The process of MALDI-TOF MS method was executed as previously outlined (Li et al., 2024a).

### Antimicrobial susceptibility testing

The susceptibility testing was performed as previously outlined using the broth microdilution method with Sensititre™Gram-negative panels (Thermo Scientific, Co., Ltd., Shanghai, China), in accordance with the guidelines recommended by the Clinical and Laboratory Standards Institute (CLSI) (Li et al., 2024a). The antimicrobial agents included in the susceptibility testing were as follows: imipenem (0.06-128 mg/L), meropenem (0.03-64 mg/L), ceftazidime-avibactam (0.03-64 mg/L), cefepime (0.06-128 mg/L), amikacin (1-128 mg/L), ceftazidime (0.25-32 mg/L), aztreonam (1-128 mg/L), ciprofloxacin (0.06-8 mg/L), cefoperazone/sulbactam (1-128 mg/L), piperacillin-tazobactam (2-256 mg/L), colistin (0.125-8 mg/L). *E. coli* ATCC 25922 was used as the reference strain.

### Plasmid conjugation experiment

Conjugation experiment was performed on the *KPC*-variant positive *P. aeruginosa* strain-*bla*_KPC-249_ (a novel *bla*_KPC-2_ variant) via the filter mating method. The rifampicin-resistant *P. aeruginosa* strain PAO1 (designated as PAO1^rifR^) was employed as the recipient strain. Initially, single colonies of the donor *P. aeruginosa* strain and the recipient PAO1^rifR^ were each picked and cultured in 4 mL of LB broth at 35°C with constant shaking for 4 hours. Subsequently, the resulting suspensions of the donor and recipient strains were gently mixed at a 1:2 ratio. For the filter mating method, the resulting mixed suspension was subsequently cultured for 24 hours at 35°C on a filter. Transconjugants were selected on a medium supplemented with 600μg/mL rifampicin and 8 μg/mL CZA, followed by screening for the presence of the *bla*_KPC_ gene via PCR with the specific primers: forward primer: 5’-*TGTGTACGCGATGGATACCG*-3’ and reverse primer: 5’-*GTTGACGCCCAATCCCTCG*-3’ (Hu et al., 2024).

### Short-read genome sequencing and analysis

The Tianamp Bacteria DNA Kit (Tiangen Biochemical Technology (Beijing) Co., Ltd.) was employed for the extraction of genomic DNA. The extracted genomic DNA was subjected to high-throughput paired-end sequencing (PE150) using the Illumina NovaSeq 6000 platform. Fastp v0.23.2 was employed for trimming raw reads, followed by assembly of the trimmed reads using Unicycler v0.5.0 under default settings. Prokka v1.14.5 was employed for gene prediction and functional annotation. Sequence typing, capsule typing, and screening of virulence factors and acquired antimicrobial resistance genes were conducted with Kleborate v2.0.1. MOB-suite v3.0.1 was employed to predicate plasmid sequences from hybrid assemblies and to determine their respective replicon types. Both the CARD and ResFinder databases were employed for detecting the presence of resistance genes (Li et al., 2024a).

### Long-read genome sequencing and analysis

The 10K SMRT Bell library was constructed with the SMRTbell™ Template Kit (v1.0). For library preparation, DNA samples qualified by electrophoretic quality assessment were fragmented to the required size using a Covaris g-TUBE. The fragmented DNA was first subjected to sequential DNA damage repair and end repair, subsequently, hairpin adapters were ligated to both 5’ and 3’ ends of the DNA fragments using DNA ligase. Purification of the resulting DNA fragments was performed using AMPure PB beads. A BluePippin system was employed for the selection of specific fragment sizes, followed by further purification of the SMRT Bell library using AMPure PB beads for concentration screening. Following the repair of DNA damage, the library was subjected to a second purification step using AMPure PB beads. The final library’s concentration and insert size distribution were determined using a Qubit fluorometer and an Agilent 2100 Bioanalyzer, respectively. Sequencing was conducted on the PacBio Sequel platform (Wu et al., 2023).

### Molecular cloning

In this study, *bla*_KPC-249_ gene fragments carrying wild-type promoter were amplified from the genomic DNA of *P. aeruginosa* by PCR. The amplification reaction was performed using specific primer pairs pro398-BamHI-R (5 ‘ -CGACTCTAGAGGATCCAATAGATGATTTTCAGAGCCTTAC-3 ‘) and pro398-EcoRI-F (5 ‘ -CCATGATTACGAATTGTGCGGAACCCCTATTTG-3 ‘) using Phanta Flash premix reagent (Vazyme, product number P520). The amplification conditions are as follows: denaturation at 94°C for 30 seconds, annealing at 56°C for 40 seconds, and extension at 72°C for 50 seconds. Repeat the above procedure for 30 cycles, followed by a final extension at 72°C for 7 minutes. The amplified product was purified using the FastPure nucleic acid purification kit (Vazyme, product number DC301) and ligated with pHSG398 plasmid vector (Takara) to construct a recombinant plasmid. The ligation conditions for cloning are as follows: incubation at 37°C for 45 minutes, followed by treatment at 85°C for 10 seconds, and finally incubation at 4°C for 5 minutes. In this experiment, *E.coli DH5α* was used as the host strain for plasmid construction. The recombinant plasmid was introduced into *DH5α* competent cells (Vazyme, product number C502), and the plasmid was amplified by shaking at 37°C and 220 rpm. Subsequently, the bacterial solution containing the recombinant plasmid was coated on LB solid medium supplemented with 50 mg/L chloramphenicol and 50 mg/L ampicillin for positive clone screening.

### Ethical statement

This study was approved by the hospital ethics committee, approval number: 2024-117-01.

## Results

### Antimicrobial susceptibility testing

The resistance profiles of all 5 P*. aeruginosa* strains (designated as PAE1, PAE2, PAE045, PAE3, and PAE4) are presented in **Table 1**. For imipenem susceptibility, the minimum inhibitory concentrations (MICs) of strains PAE045 and PAE3 were 8 μg/mL and 16 μg/mL, respectively; in contrast, the MICs of the remaining three strains (PAE1, PAE2, PAE4) to imipenem were uniformly 2 μg/mL. Regarding meropenem, the MICs of PAE045 and PAE3 were 32 μg/mL and 16 μg/mL, respectively, while the MICs of the other three *P. aeruginosa* strains to meropenem were all ≤ 1 μg/mL. For ceftazidime-avibactam (CZA), the MIC of PAE045 exceeded 64 μg/mL, whereas the MICs of the other four *P. aeruginosa* strains to CZA were consistently 2 μg/mL.

**Table 1:** Susceptibilities of strains mentioned in the study (μg/mL).

Strains	IPM	MEM	CZA	AMK	FEP	CAZ	ATM	CIP	CSL	TZP	COL
PAE1	2	0.5	2	8	4	4	8	0.5	8	4	1
PAE2	2	1	2	8	4	8	8	0.5	8	4	1
PAE045	8	32	>64	2	>32	>32	32	>8	64	32	2
PAE3	16	16	2	8	16	4	32	8	64	8	1
PAE4	2	1	2	8	16	4	8	0.5	8	8	1
PAO1*^rifR^*^a^	1	0.5	2	2	4	2	16	0.125	8	8	0.5
PAO1^rifR^/PAE045 ^b^	8	32	>64	2	>32	>32	32	>8	64	32	1
DH5α-transconjugant^c^	0.25	0.06	8	1	2	>32	1	0.06	/	4	0.125
DH5α-*bla*_KPC249_^d^	0.25	0.06	16	1	2	>32	2	0.06	/	4	0.125

IPM, imipenem; MEM, meropenem; CZA, ceftazidime-avibactam; AMK, amikacin; FEP, cefepime; CAZ, ceftazidime; ATM, aztreonam; CIP, ciprofloxacin; CSL, cefoperazone/sulbactam; TZP, piperacillin-tazobactam; COL, colistin.

a PAO1^rifR^:a spontaneous rifampicin-resistant mutant *P. aeruginosa* PAO1strain15 (Hu et al., 2024).

b PAO1^rifR^/PAE045_:the recipient strain used for conjugation experiments.

c DH5α-transconjugant: *E. coli* DH5a was transformed by *P. aeruginosa* PAE045.

d DH5α-*bla*_KPC249_: *E. coli* DH5a was transformed by a plasmid carrying *bla_KPC-249_* gene.

### Conjugation transfer and molecular cloning results

After the conjugation transfer experiment, the minimum inhibitory concentration (MIC) of the transconjugant PAO1^rifR^/PAE045 (obtained via filter membrane transfer) against ceftazidime-avibactam increased from 1 mg/L (the MIC of the recipient strain PAO1*^rifR^* against ceftazidime-avibactam) to 64 mg/L. Polymerase chain reaction (PCR) detection results of the transconjugant confirmed that the PAO1^rifR^/PAE045 transconjugant carried the *bla*_KPC-249_ resistance gene, which mediates resistance to ceftazidime-avibactam. The DH5α-*bla*_KPC249_ of *E. coli* DH5a carrying the *bla*_KPC249_ gene showed resistance to CZA. Detailed data are presented in **Table 1**.

### The genetic surroundings of *bla*_KPC-249_ and comparison of plasmids structures

**Figure 2:**
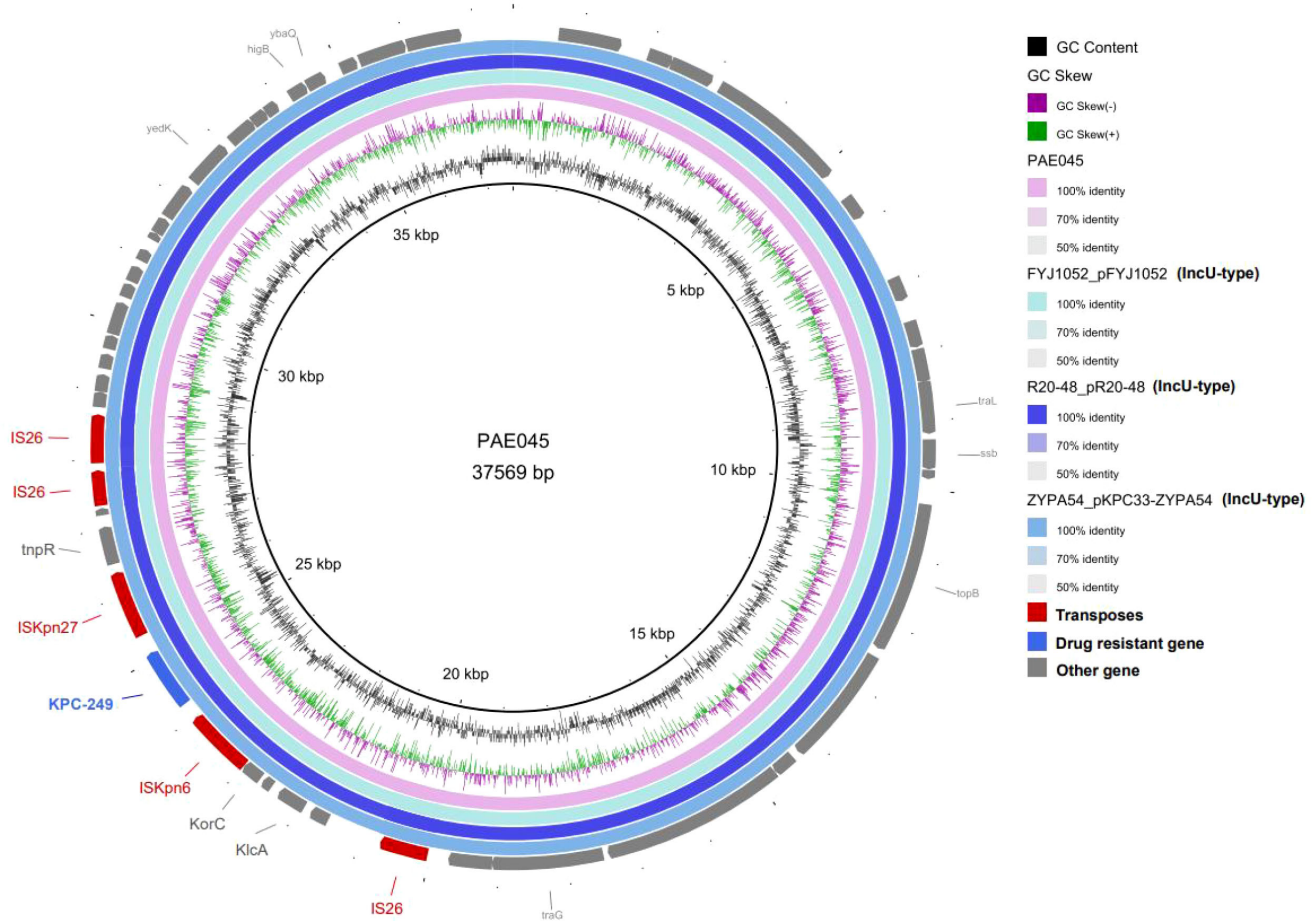
Comparative plasmid map of PAE045, pFYJ1052, pR20-48, and pKPC33-ZYPA54. From the inside to the outside: circle 1, scale; circle 2, GC content; circle 3, GC Skew; circle 4, ring diagram of PAE045; circle 5, ring diagram of pFYJ1052; circle 6, ring diagram of pR20-48; circle 7, ring diagram of pKPC33-ZYPA54; circle 8, functional classified genes.

**Figure 3:**
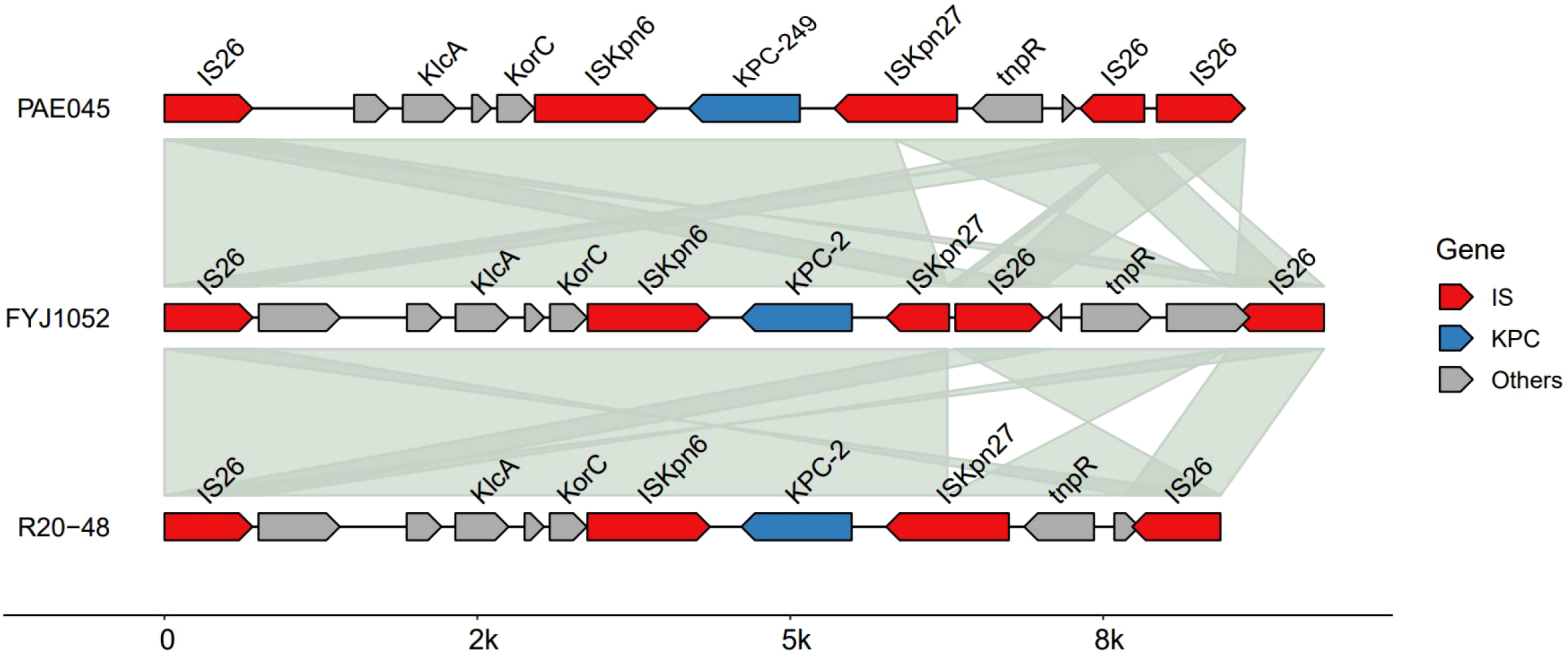
Genetic environment diagram: Comparison of the upstream and downstream gene environment of *bla*_KPC-249_ in PAE045 *P. aeruginosa* with the upstream and downstream gene environment of *bla*_KPC-2_ in FYJ1052 and R20-48.

**Figure 4:**
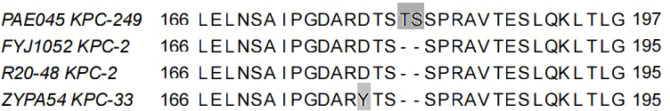
The mutation sites and amino acid sites of the *bla*_KPC-249_ gene and the *bla*_KPC-2_ gene were changed. Two amino acid sequences were added at positions 182 and 183, specifically threonine (Thr, T) at position 182 and serine (Ser, S) at position 183, respectively.

According to the whole-genome sequencing (WGS) results of this study, the *bla*_KPC-249_ gene in *P. aeruginosa* strain PAE045 was localized on a plasmid with a size of 37,569 bp, and also carried one new antibiotic resistance gene, *bla*_KPC-249_, along with five transposons, including three IS*26*, one IS*Kpn27*, and one IS*Kpn6*, confer the potential for the rapid horizontal transfer of antibiotic resistance genes. PAE045 strain harbored only a single plasmid. This plasmid belongs to an unknown subtype and is speculated to be of IncU origin, given that its homologs are IncU-type plasmids. The *bla*_KPC-249_ gene is inserted between IS*Kpn6* and IS*Kpn27*, and the plasmid is non-mobilizable. The upstream and downstream genetic environments of the *bla*_KPC-249_ gene in PAE045 were similar to those of *bla*_KPC-2_ in strains FYJ1052 and R20-48, with the flanking elements identified as IS*Kpn27* and IS*Kpn6*, respectively (as shown in **Figures 2**, **3**). Compared with *bla*_KPC-2_, *bla*_KPC-249_ was characterized by the addition of two amino acid residues at positions 182 and 183, which were threonine (Thr, T) and serine (Ser, S), respectively (**Figure 4**).

## Discussion

According to the CHINET Surveillance Network, the resistance rates of *P. aeruginosa* to common antibiotics between 2005 and 2022 were as follows: 14.3%–29% for ceftazidime, 3.3%–23% for amikacin, 23.8%–31% for imipenem, 15%–31% for ciprofloxacin, and 11.7%–34% for piperacillin-tazobactam; meanwhile, the detection rate of carbapenem-resistant *P. aeruginosa* (CRPA) ranged from 7.6% to 26.2%. The data from the CARSS Surveillance Network in China for 2022 showed that the average detection rate of CRPA was 16.6%, whereas the CRPA detection rate reported by CHINET in 2022 was 23.8%. According to data from the European Antimicrobial Resistance Surveillance Network (EARS-Net), the resistance rate of carbapenem-resistant *P. aeruginosa* (CRPA) in Europe reached 18.6% in 2023; notably, the resistance rate of CRPA strains in southern and eastern European countries was slightly higher than that in northern and western European countries. For other antibiotics, the resistance rates of *P. aeruginosa* in Europe were 18.5% for piperacillin-tazobactam, 15.7% for ceftazidime, and 9.5% for aminoglycosides (https://www.ecdc.europa.eu/sites/default/files/documents/antimicrobial-resistance-annual-epidemiological-report-EARS-Net-2023.pdf). Although the resistance rate of *P. aeruginosa* to common major antibiotics in China and Europe has decreased compared with that in 2022, difficult-to-treat *P. aeruginosa* (DTR-PAE) still poses significant challenges to clinical treatment. DTR-PAE is defined as *P. aeruginosa* strains non-susceptible to all “traditional” β-lactam antibiotics (i.e., piperacillin-tazobactam, ceftazidime, cefepime, aztreonam, meropenem, and imipenem) and fluoroquinolones (i.e., ciprofloxacin and levofloxacin) (Kadri et al., 2018). Data indicate that the 30-day mortality rate associated with DTR-PAE infections is approximately 20% (Hareza et al., 2024). The U.S. Centers for Disease Control and Prevention (CDC) reported that in 2017 alone, there were more than 32,000 cases of multidrug-resistant *P. aeruginosa* infections (Centers for Disease Control and Prevention, 2019). The clinical case in this study involved a female patient admitted to the intensive care unit (ICU). The patient was infected with carbapenem-resistant *P. aeruginosa* and received ceftazidime-avibactam (2.5 g, administered every 8 hours, Q8H) for treatment. During the treatment, a *bla*_KPC_ variant was detected in the *P. aeruginosa* isolate. Ceftazidime-avibactam serves as a crucial treatment option for patients with multidrug-resistant or difficult-to-treat *P. aeruginosa* (DTR-PAE) infections, particularly for those with severe CRPA infections, and is widely regarded as a first-line agent for the treatment of multidrug-resistant *P. aeruginosa*.

Under the selective pressure of antibiotics, the bacteria have corresponding drug resistance mutations. The resistance of *P.aeruginosa* to ceftazidime-avibactam is low. According to data from the CHINET Antimicrobial Resistance Surveillance Network, the resistance rate of *P. aeruginosa* to ceftazidime-avibactam ranged from 6.3% to 11.1% from 2005 to 2022. Notably, in 2024, this resistance rate was recorded at 5.8% (https://www.chinets.com/Data/AntibioticDrugFast). A multicenter study conducted in Italy reported that the resistance rate of *P. aeruginosa* to ceftazidime-avibactam reached 18.3%. Additionally, relevant data indicated that following treatment of *P. aeruginosa* with ceftazidime-avibactam, the resistance rate of the bacterium to this antimicrobial agent increased from 0% to 13% (Shah et al., 2025). Existing research suggests that the primary resistance mechanisms of *P. aeruginosa* to ceftazidime-avibactam include point mutations in β-lactamase genes, structural modifications of β-lactamase enzymes, and increased expression of efflux pumps (Berrazeg et al., 2015). Among these mechanisms, point mutations in β-lactamase genes have emerged as the primary factor contributing to ceftazidime-avibactam resistance in *P. aeruginosa (*Li et al., 2024b). The present study demonstrated that within 12 days of ceftazidime-avibactam treatment, ceftazidime-avibactam evolved from being sensitive (MIC = 2 mg/L) to resistant (MIC > 64 mg/L). However, 29 days after ceftazidime-avibactam treatment, *P. aeruginosa* returned to the sensitivity of ceftazidime-avibactam (MIC = 2 mg/L). Sequencing results revealed that the ceftazidime-avibactam resistance observed during this period was attributed to the production of a novel *bla*_KPC-2_ mutant, designated as *bla*_KPC-249_. Compared with the wild-type *bla*_KPC-2_ gene, *bla*_KPC-249_ harbored two insertion mutations, which resulted in the addition of a threonine residue at position 182 and a serine residue at position 183. These mutation sites are consistent with those identified in the *KPC* variants *bla*_KPC-104_ and *bla*_KPC-106_, which have been reported in the literature to mediate ceftazidime-avibactam resistance in *Klebsiella pneumoniae*. Nevertheless, the mutation sites of *bla*_KPC-249_ differ from those of other *KPC* variants that also confer ceftazidime-avibactam resistance (Hobson et al., 2022). P*. aeruginosa* strains carrying the *bla*_KPC-249_ gene exhibit significant differences in resistance phenotypes to various antibiotics compared with *P. aeruginosa* strains carrying other *KPC* subtypes. Specifically, the MIC value of imipenem against *P. aeruginosa* carrying *bla*_KPC-249_ is not substantially elevated (Hu et al., 2024; Zhang et al., 2024). Meanwhile, in comparison with *Klebsiella pneumoniae* strains carrying other *KPC* subtypes, the MIC value of imipenem against *P. aeruginosa* carrying *bla*_KPC-249_ does not show a significant increase, whereas the MIC value of imipenem against *Klebsiella pneumoniae* carrying other *KPC* subtypes is markedly elevated (Yang et al., 2022). Studies have indicated that serine and threonine residues are among the key catalytic residues involved in the inhibition of avibactam. These residues provide protons to the sulfate nitrogen (N6) of avibactam, thereby facilitating the acylation process (Lahiri et al., 2016). Notably, molecular cloning experiments have further confirmed that the *bla*_KPC-249_ mutant exhibits reduced sensitivity to ceftazidime-avibactam. This represents the first report of the *bla*_KPC-249_ gene in *P. aeruginosa*, as well as the first documentation of its involvement in mediating ceftazidime-avibactam resistance. Furthermore, insights from the case analyzed in this study suggest that the continuous administration of ceftazidime-avibactam (particularly at full doses and for the complete recommended course) is highly likely to reduce the sensitivity of *P. aeruginosa* to this antimicrobial agent and is also a key condition for the emergence of *KPC* variants. Therefore, during the course of clinical treatment, it is recommended to monitor the MIC value of *P. aeruginosa* to ceftazidime-avibactam daily. If ceftazidime-avibactam-resistant strains are detected, the administration of ceftazidime-avibactam should be immediately discontinued, and alternative antibiotics should be initiated. Currently, for the treatment of multidrug-resistant (MDR) *P. aeruginosa*, particularly DTR-PAE infections, antimicrobial agents such as doripenem, ceftaroline fosamil, and plazomicin can be employed (Abdul-Mutakabbir et al., 2019; Losito et al., 2022; Lee et al., 2023). Additionally, murepavadin, colistin, and polymyxin B also exhibit potent antimicrobial activity against *P. aeruginosa (*Wei et al., 2024). Importantly, these agents often demonstrate favorable synergistic effects when combined with β-lactam antibiotics (e.g., ceftazidime-avibactam) and aminoglycoside antibiotics (e.g., amikacin) (Yang et al., 2025).

In addition, the *bla*_KPC-249_ gene was identified to be located on a plasmid with an approximate size of 37.6 kbp. Insertion sequences IS*Kpn6* and IS*Kpn27* were detected upstream and downstream, respectively. This genetic environment is consistent with that of the *bla*_KPC-2_ gene and exhibits similarity to the genetic environment of *bla*_KPC_ subtypes observed in other ceftazidime-avibactam-resistant *P. aeruginosa* strains (Tu et al., 2022; Hu et al., 2024). This finding suggests that the plasmid-mediated transmission of resistance associated with this novel subtype does not differ significantly from that of known subtypes. Multilocus sequence typing (MLST) results indicated that the PAE045 strain belongs to the ST463 clone. Previous studies have demonstrated that ST463 clones exhibit higher resistance to anti-*Pseudomonas* agents compared with non-ST463 carbapenem-resistant *P. aeruginosa* (CRPA) isolates; additionally, ST463 clones are associated with a significantly higher mortality rate (Hu H. et al., 2021). Carbapenem-resistant *P. aeruginosa* (CRPA) is one of the most prevalent pathogens in healthcare-associated infections, posing a severe challenge to global public health. Worldwide, significant variations exist in the distribution of carbapenemases and the associated sequence types (STs) among clinically isolated CRPA strains. In most geographical regions outside China, B1 metallo-β-lactamase is the predominant carbapenemase type, primarily detected in high-risk clones, such as ST235 and ST111 (Reyes et al., 2023). Notably, clinically isolated CRPA strains in China exhibit distinct genomic characteristics. A multicenter epidemiological survey revealed that 40.4% of CRPA strains in China carry the *bla*_KPC-2_ gene, among which 70.9% of *bla*_KPC-2_-producing *P. aeruginosa* (KPC-2-PA) strains belong to the ST463 clone (Zhu et al., 2021). Therefore, the prevalence of the *bla*_KPC-2_ gene may represent an important factor contributing to the successful evolutionary expansion of the ST463 clone in China. Given these observations, it is imperative to strengthen surveillance measures to prevent the spread of high-risk clones carrying *bla*_KPC_ genes—particularly ST463 *P. aeruginosa*, which may emerge as a significant public health threat in the future. Furthermore, due to the production of various carbapenemases, the failure rate of β-lactam agents (e.g., ceftazidime-avibactam) in treating *P. aeruginosa* infections has increased. Consequently, drug resistance surveillance and carbapenemase type detection have significant value in the treatment and prognosis of *P.aeruginosa* infections.

## Conclusion

In this study, a strain of ceftazidime-avibactam-resistant *P. aeruginosa* was isolated from the blood of a patient with a pulmonary infection who had been treated with ceftazidime-avibactam. Whole-genome sequencing (WGS) was employed to analyze the sequence type (ST) clone, drug resistance genes, and plasmid genetic environment of this *P. aeruginosa* strain. Notably, the *bla*_KPC-249_ gene was identified in ceftazidime-avibactam-resistant *P. aeruginosa* for the first time. The *bla*_KPC-249_ gene subtype was first discovered by our group and submitted to the National Center for Biotechnology Information (NCBI) database. It is a new *bla*_KPC_ variant that mediates ceftazidime-avibactam resistance in *P. aeruginosa* and can successfully cause horizontal dissemination. This study presents a novel research perspective for investigating the clinical mechanism of drug resistance to ceftazidime-avibactam in *P. aeruginosa*.

## Data Availability

The datasets presented in this study can be found in online repositories. The names of the repository/repositories and accession number(s) can be found in the article/supplementary material.
